# Supplementary material for: SIgA structures bound to Streptococcus pyogenes M4 and human CD89 provide insights into host-pathogen interactions
Source: Nat Commun. 2023 Oct 23;14:6726. doi: 10.1038/s41467-023-42469-y (PMC10593759; doi:10.1038/s41467-023-42469-y)
Supplement: Supplementary file 1 — Supplementary Information [file 41467_2023_42469_MOESM1_ESM.pdf]

SUPPLEMENTARY INFORMATION FOR

SIgA structures bound to *Streptococcus pyogenes* M4 and human CD89 provide insights into host-pathogen interactions

Qianqiao Liu[1] and Beth M. Stadtmueller[1, 2, 3*]

[1]Department of Biochemistry, University of Illinois Urbana-Champaign, Urbana, Illinois 61801 USA

[2]Department of Biomedical and Translational Sciences, Carle Illinois College of Medicine, University of Illinois Urbana-Champaign, Urbana, Illinois 61801 USA

[3] Carl R. Woese Institute for Genomic Biology, University of Illinois, Urbana, Illinois 61801 USA

*address correspondence to bethms@illinois.edu

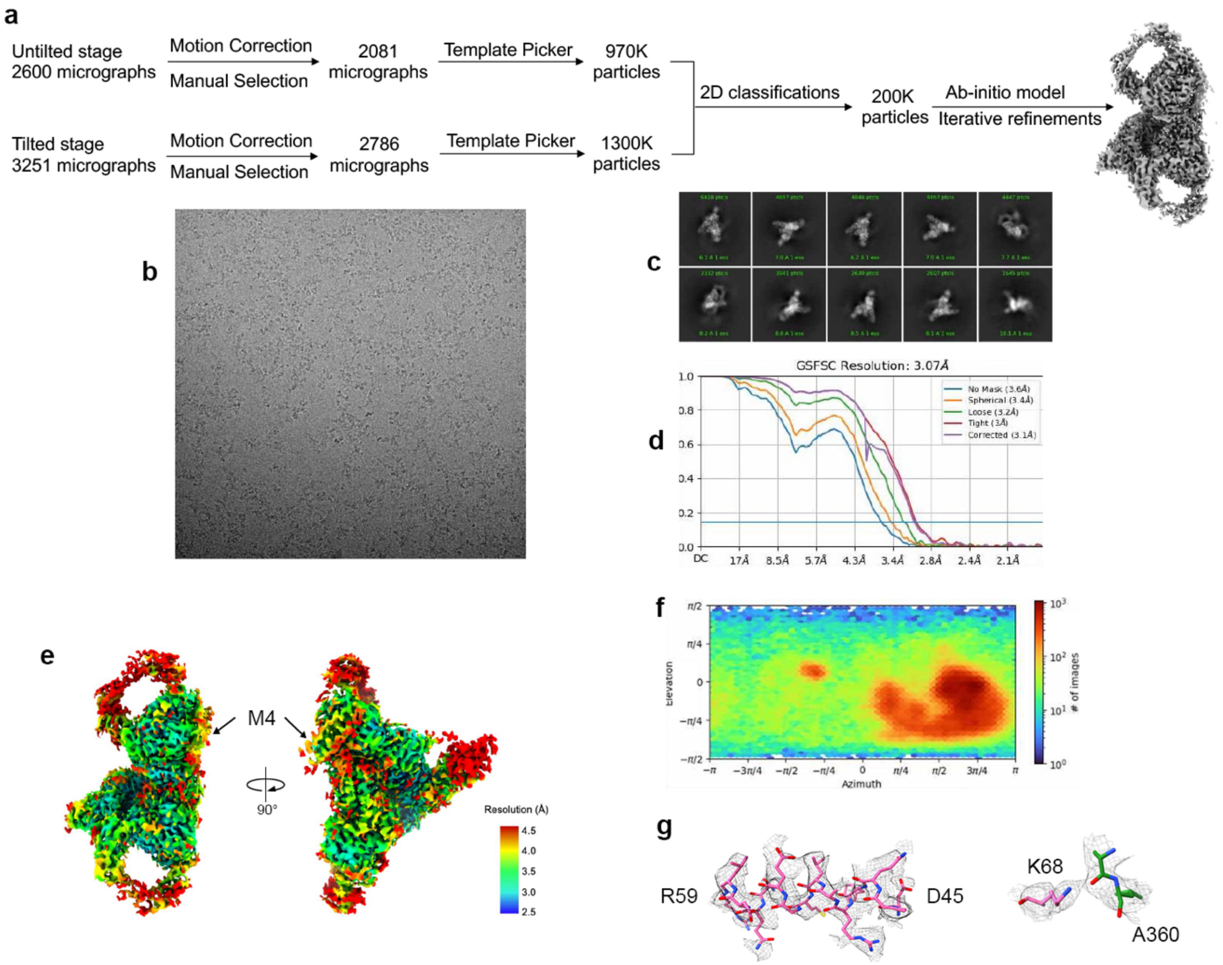

Supplementary Figure 1. CryoEM data collection and CryoSPARC processing pipeline. (a) Schematic summary of data processing pipeline of SIgA-M4 complex in CryoSPARC; (b) representative micrograph; (c) representative 2D class averages; (d) FSC curve for the final reconstruction with reported resolution at FSC=0.143 shown by the blue horizontal line. (e) Local resolution map calculated by cryoSPARC and colored in Chimera. (f) Angular distribution of particles used in the final reconstruction. (g) Density from M4 helix-a residues 45-49 and interactions between M4 K68 and SIgA A360 contoured to 3σ and carved at 2 Å cutoff.

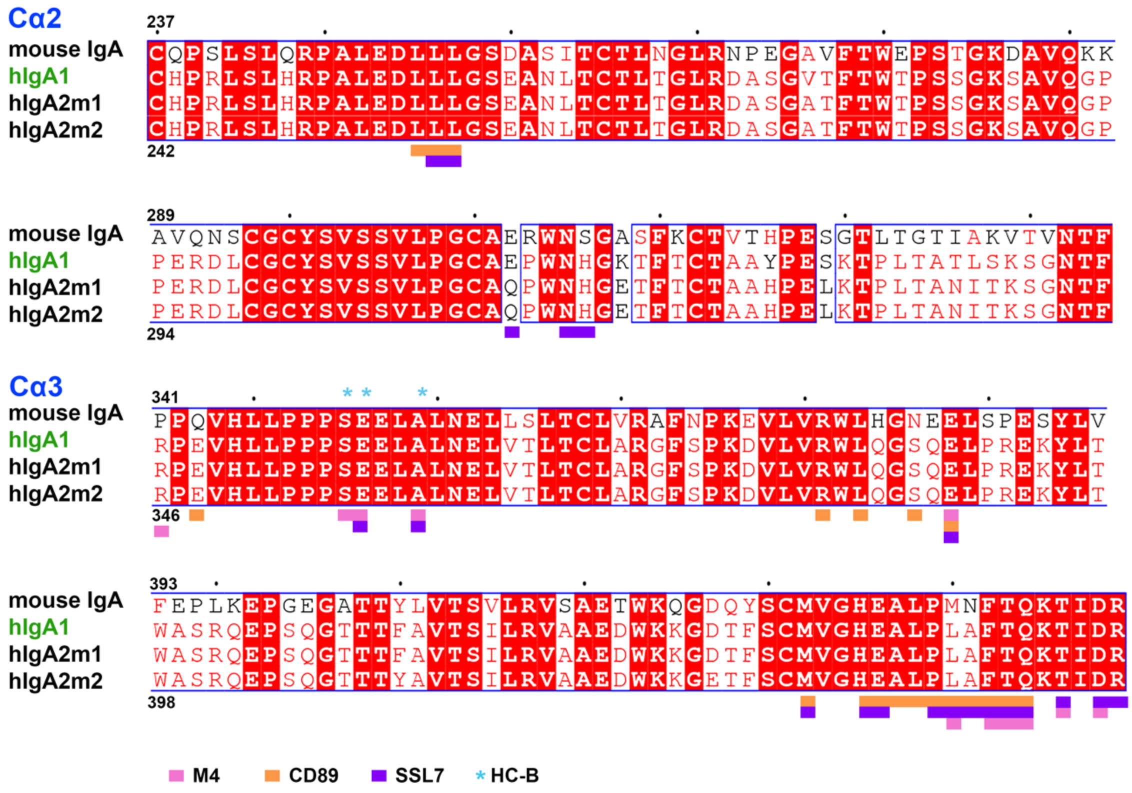

Supplementary Figure 2. Sequence alignment of human and mouse IgA heavy chains. Sequence alignment including human IgA subtypes (hIgA1, hIgA2m1 and hIgA2m2) and mouse IgA. Identical residues are colored in red. Each square denotes an interfacing residue described in the main text. Squares are colored according to the key at the bottom of the figure, blue asterisks indicate that the residue is on heavy chain-B.

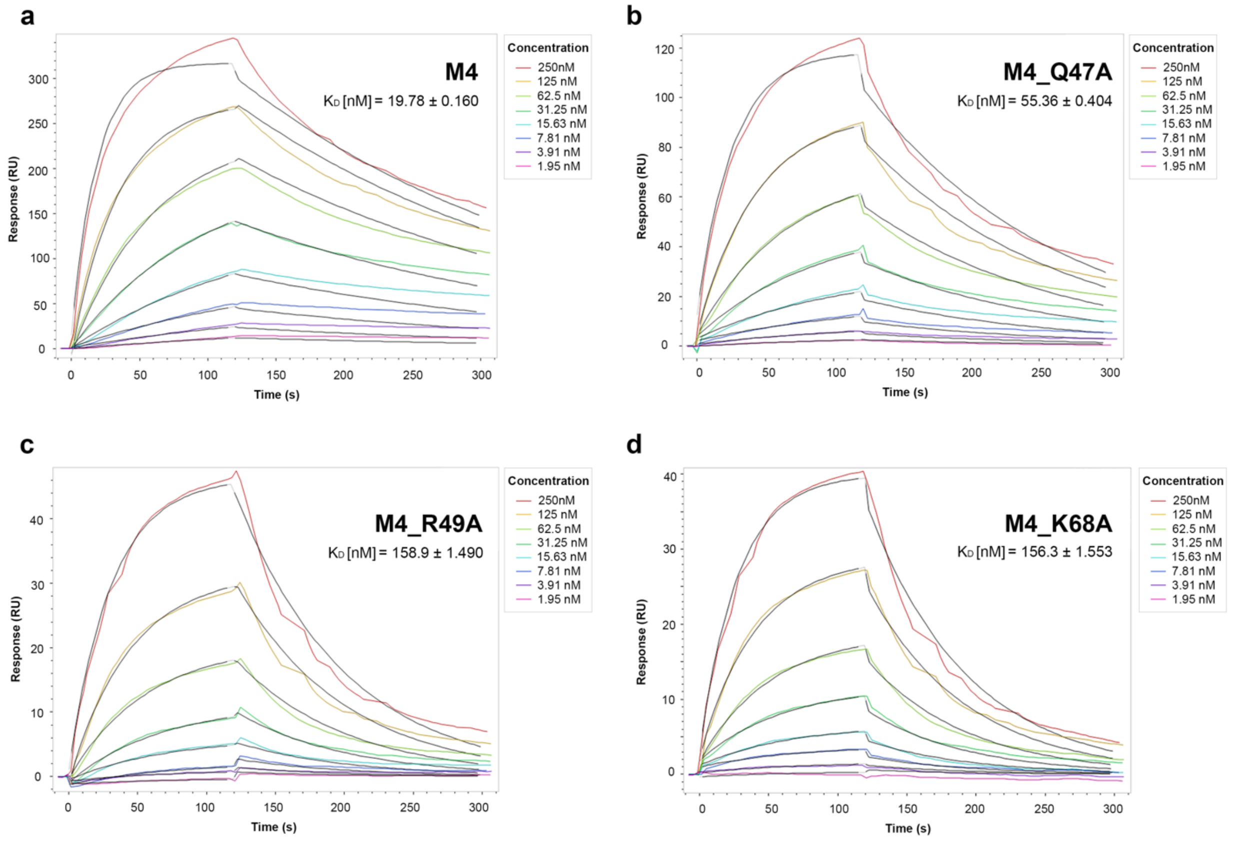

Supplementary Figure 3. M4 binding analysis by surface plasmon resonance (SPR). Sensorgrams showing the response of M4 (a) and individual M4 point mutants, Q47A (b), R49A (c), and K68A (d) binding to immobilized hSIgA1. All data were collected on a surface immobilized with 0.2uM hSIgA1 and all analytes were tested with a two-fold dilution series starting from 0.25uM. The experimental curves were fit with 1:1 binding models, shown in black. A complete kinetics analysis can be found in Supplementary Table 2. Source data are provided in the Source Data file.

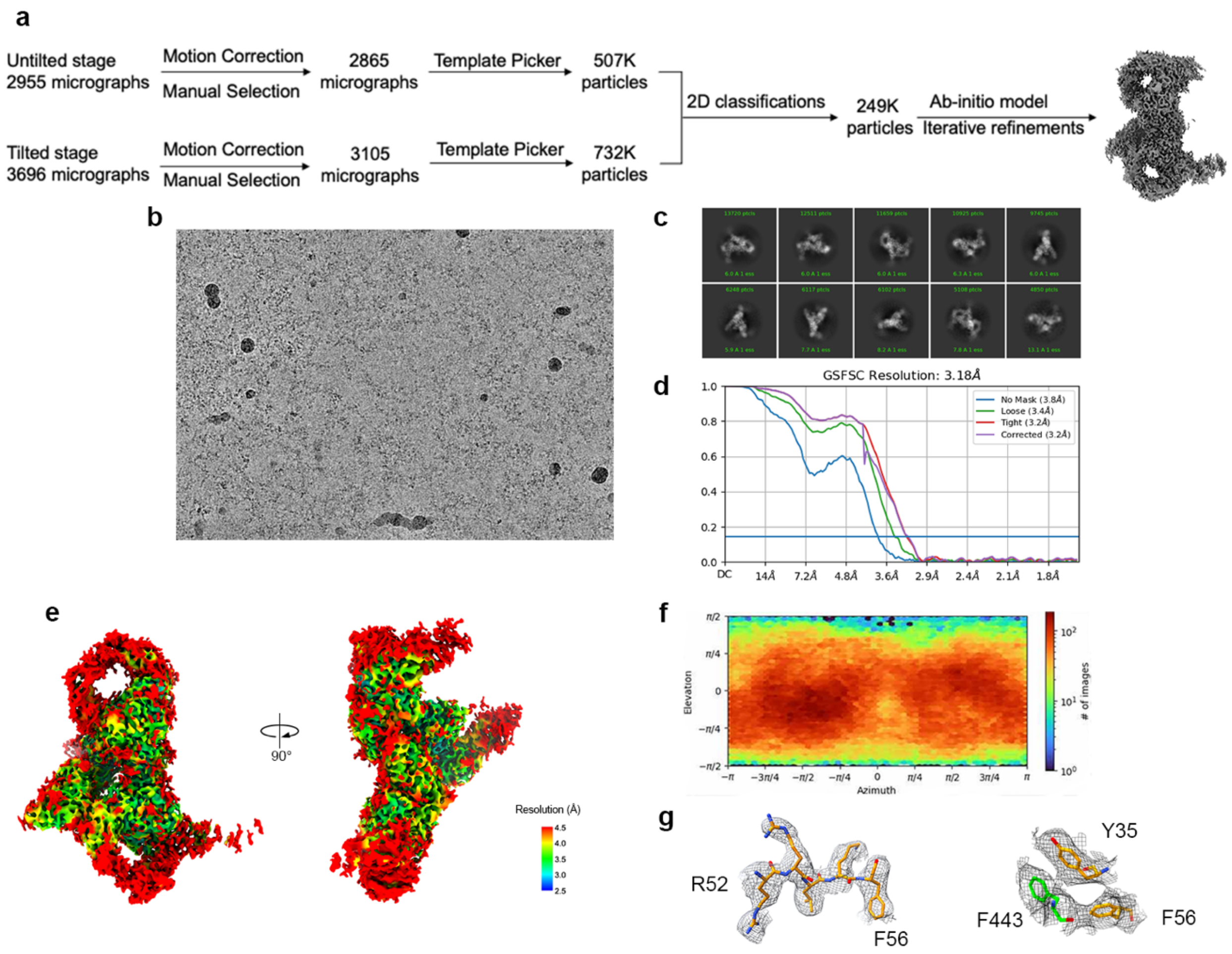

Supplementary Figure 4. SIgA-CD89 CryoEM data collection and CryoSPARC processing pipeline. (a) Schematic summary of data processing pipeline of SIgA-CD89 complex in CryoSPARC; (b) representative micrograph; (c) representative 2D class averages. (d) FSC curve for the final reconstruction with reported resolution at FSC=0.143 shown by the blue horizontal line. (e) Local resolution map calculated by cryoSPARC and colored in Chimera. (f) Angular distribution of particles used in the final reconstruction. (g) Density from CD89 residues 52-56 and part of the hydrophobic core of the SIgA-CD89 interface contoured to 3σ and carved at 2 Å cutoff.

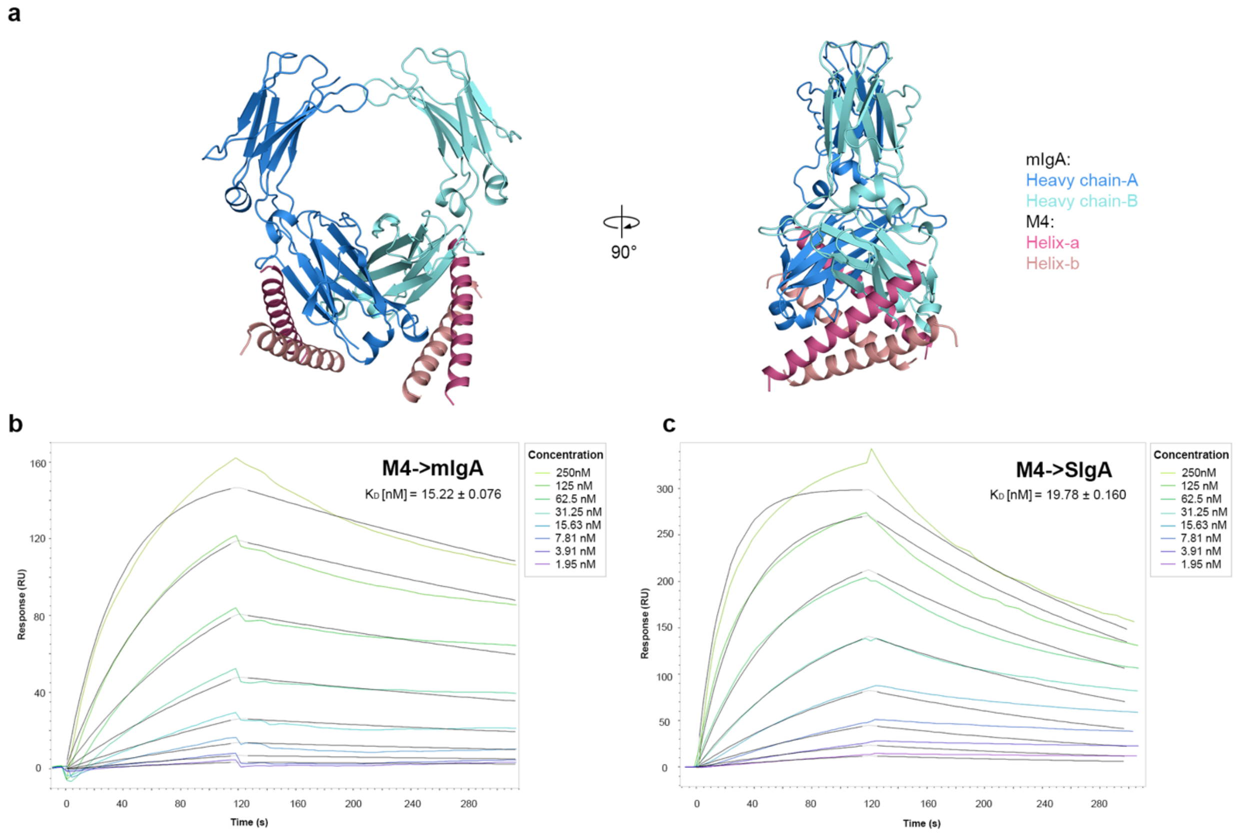

Supplementary Figure 5. M4 binding to mIgA and SIgA. (a) Model of M4 (pink) bound mIgA (blue) shown in two orientations. The model was made by aligning two copies of HC-A (bound to M4) from the M4-SIgA structure to the two IgA heavy chains from the Fcα-CD89 crystal structure (PDB code 1OW0 with CD89 removed). M4-HC-A contacts (within 4Å) observed in the model are equivalent to those observed in the SIgA-M4 structure; however, Tp residues are not ordered 1OW0 and thus we cannot completely rule out the possibility that differences between the mIgA-M4 and SIgA-M4 interfaces exist. For example, in SIgA-M4 HC-A K454 is ~ 4.5Å from M4 helix b residues D67 and E70 and could contribute to electrostatic interactions. (b) SPR Sensorgrams showing the response of M4 binding to immobilized hmIgA1. (c) SPR Sensorgrams showing the response of M4 binding to immobilized hSIgA1. Data were collected on a surface immobilized with 0.2uM hSIgA1 or 0.2uM hmIgA1 and all analytes were tested with a two-fold dilution series starting from 0.25uM. The experimental curves were fit using a 1:1 binding model, shown in black. A complete kinetics analysis can be found in Supplementary Table 2. Source data are provided in the Source Data file.

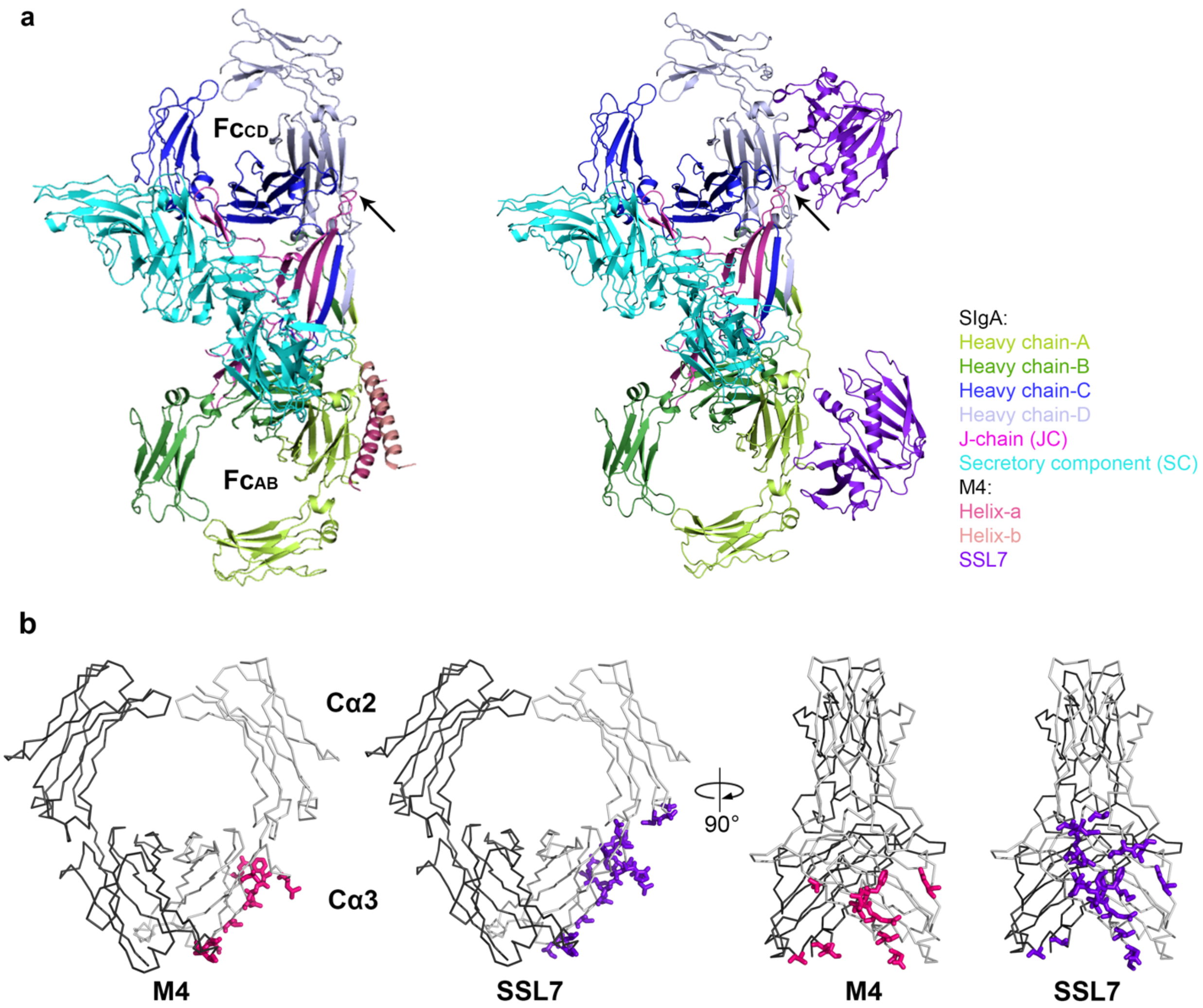

Supplementary Figure 6. Comparison of M4 and SSL7 binding to SIgA. (a) The structure of M4-SIgA and model of SSL7 bound to SIgA. The SSL7-SIgA model was built by aligning the cocrystal structure of an SSL7-Fcα complex (PDB: 2QEJ) to both copies of SIgA Fcα. M4 only binds at Fc_{AB}, as shown in Fig. 5 and the second site (Fc_{CD}) is occluded by JC (magenta) whereas SSL7 (purple) bound to the second site (Fc_{CD}) is not overlapping with any part of SIgA, indicating that binding is feasible. The JC loop that occluded M4 binding is indicated by an arrow. (b) Comparison of M4 and SSL7 binding sites on Fcα. Monomeric Fcαs (PDB code: 1OW0 with CD89 removed) are shown as ribbons and binding residues are shown as stick representations. The 11 residues bound by M4 are shown in pink and the 20 residues bound by SSL7 are shown in purple. Nine Cα3 residues are bound by both M4 and SSL7 including: E357 (HC-B), A360 (HC-B), E389, L441, F443, T444, Q445, T447, D449. The other 11 residues bound by SSL7 interface are: L257, L258, E313, N316, H317, M433, H436, E437, P440, A442, R450.

Supplementary Table 1. Cryo-EM data collection, refinement, and validation statistics

	#1 SIgA:M4 (EMDB-40568) (PDB 8SKV)	#2 SIgA:CD89 (EMDB-40567) (PDB 8SKU)
**Data collection and processing**		
Magnification	130K	165K
Voltage (kV)	300	300
Electron exposure (e–/Å^2)	50	71
Defocus range (μm)	-0.8 to -2.0	-0.8 to -2.0
Pixel size (Å)	0.95	0.82
Symmetry imposed	C1	C1
Initial particle images (no.)	2270K	1240K
Final particle images (no.)	200K	249K
Map resolution (Å)	3.07	3.18
FSC threshold	0.143	0.143
Map resolution range (Å)	2-5	2-5
**Refinement**		
Initial model used (PDB code)	6ue7	6ue7, 1ow0
Model resolution (Å)	3.3	3.7
FSC threshold	0.5	0.5
Map sharpening *B* factor (Å^2)	185	170
Model composition		
Non-hydrogen atoms	12220	14476
Protein residues	1632	1951
Ligands	NAG:15	NAG:12
B factors (Å^2)		
Protein	40.39	52.26
Ligand	67.15	74.18
R.m.s. deviations		
Bond lengths (Å)	0.002	0.005
Bond angles (°)	0.544	0.642
Validation		
MolProbity score	1.67	1.83
Clashscore	5.26	7.13
Poor rotamers (%)	0.32	0.2
Ramachandran plot		
Favored (%)	94.32	93.15
Allowed (%)	5.77	6.85
Disallowed (%)	0	0

Supplementary Table 2. Kinetic analysis of SPR experiments

Ligand	Analyte	K_{on} [$M^{-1}s^{-1}$]	K_{off} [s^{-1}]	K_D [nM]
SIgA	M4	2.009e5 ± 1032	3.974e-3 ± 2.477e-5	19.78 ± 0.160
SIgA	M4_Q47A	1.273e5 ± 761.3	7.044e-3 ± 2.953e-5	55.36 ± 0.404
SIgA	M4_R49A	7.937e4 ± 692.2	1.261e-2 ± 4.353e-5	158.9 ± 1.490
SIgA	M4_K68A	8.860e4 ± 819.3	1.384e-2 ± 5.052e-5	156.3 ± 1.553
mIgA	M4	1.012e5 ± 262.6	1.541e-3 ± 6.591e-6	15.22 ± 0.076
