# Supplementary material for: SIgA structures bound to Streptococcus pyogenes M4 and human CD89 provide insights into host-pathogen interactions
Source: Nat Commun. 2023 Oct 23;14:6726. doi: 10.1038/s41467-023-42469-y (PMC10593759; doi:10.1038/s41467-023-42469-y)
Supplement: Supplementary file 3 — Reporting Summary [file 41467_2023_42469_MOESM3_ESM.pdf]

nature portfolio

Corresponding author(s): Beth Stadtmuller

Last updated by author(s): Sep 22, 2023

Reporting Summary

Nature Portfolio wishes to improve the reproducibility of the work that we publish. This form provides structure for consistency and transparency in reporting. For further information on Nature Portfolio policies, see our Editorial Policies and the Editorial Policy Checklist.

Statistics

For all statistical analyses, confirm that the following items are present in the figure legend, table legend, main text, or Methods section.

n/a	Confirmed	
☒	☐	The exact sample size (*n*) for each experimental group/condition, given as a discrete number and unit of measurement
☒	☐	A statement on whether measurements were taken from distinct samples or whether the same sample was measured repeatedly
☒	☐	The statistical test(s) used AND whether they are one- or two-sided<br>*Only common tests should be described solely by name; describe more complex techniques in the Methods section.*
☒	☐	A description of all covariates tested
☒	☐	A description of any assumptions or corrections, such as tests of normality and adjustment for multiple comparisons
☒	☐	A full description of the statistical parameters including central tendency (e.g. means) or other basic estimates (e.g. regression coefficient) AND variation (e.g. standard deviation) or associated estimates of uncertainty (e.g. confidence intervals)
☒	☐	For null hypothesis testing, the test statistic (e.g. *F*, *t*, *r*) with confidence intervals, effect sizes, degrees of freedom and *P* value noted<br>*Give P values as exact values whenever suitable.*
☒	☐	For Bayesian analysis, information on the choice of priors and Markov chain Monte Carlo settings
☒	☐	For hierarchical and complex designs, identification of the appropriate level for tests and full reporting of outcomes
☒	☐	Estimates of effect sizes (e.g. Cohen's *d*, Pearson's *r*), indicating how they were calculated

Our web collection on statistics for biologists contains articles on many of the points above.

Software and code

Policy information about availability of computer code

Data collection	Cryo-EM data were collected using EPU 2.10 and SerialEM 4.0. SPR data were collected using Sierra SPR Control software.
Data analysis	Cryo-EM data were processed with CryoSPARC v 3.2.0 and v 4.1.2. Structure building was done using Coot 0.8.92 and PHENIX 1.20.1. Structure analysis was done in UCSF Chimera 1.15 and Pymol 2.5.1. AlphaFold2-multimer was used to predict the full length M4 dimer structure and ColabFold 1.5.2 was used to generate the prediction. SPR data were plotted using Bruker Analyzer R4 and Microsoft Excel. The sequence alignment was done using ClustalOmega and the aligment figure was made using ESPript3.

For manuscripts utilizing custom algorithms or software that are central to the research but not yet described in published literature, software must be made available to editors and reviewers. We strongly encourage code deposition in a community repository (e.g. GitHub). See the Nature Portfolio guidelines for submitting code & software for further information.

Data

Policy information about availability of data

All manuscripts must include a data availability statement. This statement should provide the following information, where applicable:

- Accession codes, unique identifiers, or web links for publicly available datasets
- A description of any restrictions on data availability
- For clinical datasets or third party data, please ensure that the statement adheres to our policy

The Cryo-EM density maps have been deposited in the EM databank (www.ebi.ac.uk/emdb) with the accession codes 40568, and 40567 for M4-SIgA and CD89-SIgA structures, respectively, and the refined coordinates have been deposited in the Protein Data Bank (www.rcsb.org) with accession codes 8SKV (M4-SIgA) and 8SKU (CD89-SIgA). Human SIgA structure (PDB code: 6UE7) and CD89-Fca structure (PDB code: 1OW0) were used as initial models for structure building and modeling. SSL7- Fca structure (PDB code: 2QEJ) was used for modeling in Fig. S6. The SPR data generated in this study are provided in the Source Data file.

Research involving human participants, their data, or biological material

Policy information about studies with human participants or human data. See also policy information about sex, gender (identity/presentation), and sexual orientation and race, ethnicity and racism.

Reporting on sex and gender	Human secretory IgA used in this study was recombinantly expressed and purified in HEK Expi293F cell line, so this section is not applicable to our study.
Reporting on race, ethnicity, or other socially relevant groupings	Human secretory IgA used in this study was recombinantly expressed and purified in HEK Expi293F cell line, so this section is not applicable to our study.
Population characteristics	Human secretory IgA used in this study was recombinantly expressed and purified in HEK Expi293F cell line, so this section is not applicable to our study.
Recruitment	Human secretory IgA used in this study was recombinantly expressed and purified in HEK Expi293F cell line, so this section is not applicable to our study.
Ethics oversight	Human secretory IgA used in this study was recombinantly expressed and purified in HEK Expi293F cell line, so this section is not applicable to our study.

Note that full information on the approval of the study protocol must also be provided in the manuscript.

Field-specific reporting

Please select the one below that is the best fit for your research. If you are not sure, read the appropriate sections before making your selection.

☒ Life sciences ☐ Behavioural & social sciences ☐ Ecological, evolutionary & environmental sciences

For a reference copy of the document with all sections, see nature.com/documents/nr-reporting-summary-flat.pdf

Life sciences study design

All studies must disclose on these points even when the disclosure is negative.

Sample size	SIgA-M4:5851 micrographs were initially collected, a final set of 200K particles was used for 3D reconstruction.<br>SIgA-CD89: 6651 micrographs were initially collected, a final set of 249K particles was used for 3D reconstruction.
Data exclusions	Cryo-EM micrographs with thick ice and low CTF-estimates were excluded in the data processing step as it is standardized procedure.
Replication	SPR experiments were repeated twice successfully. For cryo-EM, see "Randomization" section below as replication and randomization are closely related procedures in cryo-EM structure determination.
Randomization	The dataset was randomly split into two half-sets and refined independently in cryoSPARC, producing two half-maps. The reported resolution was calculated from the Fourier Shell Correlation between the two half-maps at the 0.143 threshold criteria, indicating that the resolution is reproducible from this dataset.
Blinding	Blinding was not applicable to this study, because the splitting of data to half-sets was performed automatically by the processing software (CryoSPARC).

Reporting for specific materials, systems and methods

We require information from authors about some types of materials, experimental systems and methods used in many studies. Here, indicate whether each material, system or method listed is relevant to your study. If you are not sure if a list item applies to your research, read the appropriate section before selecting a response.

Materials & experimental systems

n/a	Involved in the study
☒	☐ Antibodies
☐	☒ Eukaryotic cell lines
☒	☐ Palaeontology and archaeology
☒	☐ Animals and other organisms
☒	☐ Clinical data
☒	☐ Dual use research of concern
☒	☐ Plants

Methods

n/a	Involved in the study
☒	☐ ChIP-seq
☒	☐ Flow cytometry
☒	☐ MRI-based neuroimaging

Eukaryotic cell lines

Policy information about cell lines and Sex and Gender in Research

Cell line source(s)	Expi293F cells (purchased from Gibco: A14527) were used to express human SIgA and CD89 proteins.
Authentication	Expi293F cells were used as received and not authenticated.
Mycoplasma contamination	All cell lines tested negative for mycoplasma contamination.
Commonly misidentified lines (See ICLAC register)	No misidentified cell line was used in the study.
